# First-Ever Stroke Outcomes in Patients with Atrial Fibrillation: A Retrospective Cross-Sectional Study

**DOI:** 10.3390/medicines12030018

**Published:** 2025-07-24

**Authors:** Ivanka Maduna, Dorotea Vidaković, Petra Črnac, Christian Saleh, Hrvoje Budinčević

**Affiliations:** 1 Faculty of Medicine Osijek, J.J. Strossmayer University of Osijek, 31000 Osijek, Croatia; ivankamadun@gmail.com; 2 Health Center of Osijek-Baranja County, 31000 Osijek, Croatia; 3 Department of Neurology, National Memorial Hospital of Vukovar, 32000 Vukovar, Croatia; 4 Department of Neurology, Sveti Duh University Hospital, 10000 Zagreb, Croatia; 5 University of Basel, 4001 Basel, Switzerland; chs12us75010@yahoo.com; 6 Department of Psychiatry and Neurology, Faculty of Dental Medicine and Health, J.J. Strossmayer University of Osijek, 31000 Osijek, Croatia

**Keywords:** acetylsalicylic acid, atrial fibrillation, stroke, warfarin

## Abstract

**Background/Objectives**: Atrial fibrillation (AF) is the most significant modifying risk factor for the development of cardioembolic stroke, which is associated with worse outcomes and higher intrahospital mortality compared to other types of ischemic stroke. Antithrombotic medications are administered as prophylactic treatment in patients with a risk of stroke. The aim of this study was to determine outcome measures in patients with first-ever ischemic stroke and AF regarding prior antithrombotic therapy. **Methods**: We collected data on stroke risk factors, CHADS_2_ score, and international normalized ratio (INR) value in the context of warfarin therapy, as well as data related to localization, stroke severity, and functional outcome at discharge. **Results**: A total of 754 subjects with first-ever ischemic stroke and AF were included in this cross-sectional study (122 on warfarin, 210 on acetylsalicylic acid, and 422 without prior antithrombotic therapy). The diagnosis of AF was previously unknown in 31% of the subjects. Stroke risk factors (arterial hypertension, hyperlipidemia, diabetes mellitus, and cardiomyopathy) were significantly lower in the group without prior antithrombotic therapy. The anticoagulant group was significantly younger (*p* = 0.001). Overall, 45.4% of subjects with a previously known AF event and a high risk of developing stroke received anticoagulant therapy. Participants on warfarin had a significantly better functional outcome than those on antiplatelet therapy or without prior antithrombotic therapy (median mRS 4 vs. 5 vs. 5; *p* = 0.025) and lower NIHSS scores, although the difference was not statistically significant (median 10 vs. 12 vs. 12; *p* = 0.09). There was no difference between stroke localization among groups (*p* = 0.116). **Conclusions**: Our study showed that, in our cohort, first-ever ischemic stroke due to AF was more common in women. Subjects on prior anticoagulant therapy had more favorable outcomes at discharge.

## 1. Introduction

Atrial fibrillation (AF) is one of the most significant risk factors for ischemic stroke, particularly cardioembolic stroke, which is associated with higher mortality and greater disability compared to other stroke subtypes [1]. Tracz et al. showed that post-stroke patients with AF have a more severe prognosis with a higher mortality rate after ischemic stroke than patients with sinus rhythm, without an independent negative effect on the long-term outcome after stroke [2]. Clinically manifest AF has been shown to increase stroke risk three- to five-fold [3], making stroke prevention through antithrombotic therapy essential [4]. Stroke prevention in patients with AF relies on risk stratification using the CHADS_2_ and CHA_2_DS_2_-VASc scoring systems [5]. Anticoagulation is recommended for men with a CHA_2_DS_2_-VASc score of 2 points or higher and for women with a CHA_2_DS_2_-VASc score of 3 points or higher. Lower-risk patients require individualized evaluation. The prophylactic use of anticoagulant therapy in patients with AF without a prior stroke or transient ischemic attack (TIA) is based on individualized risk assessment. Additional risk factors not included in the CHA_2_DS_2_-VASc score may influence the decision. These include elevated biomarkers (NT-proBNP, troponins), echocardiographic findings, genetic predisposition to thrombosis, chronic inflammatory diseases, chronic kidney disease, and a tendency for prolonged episodes of atrial fibrillation [5,6,7,8]. A meta-analysis by Hart et al. has demonstrated the superior prophylactic efficacy of anticoagulant therapy compared to antiplatelet therapy [9]. Previous studies that included consecutive ischemic strokes in AF patients showed an association between prior anticoagulation and improved ischemic stroke outcomes [10,11,12]. Although Direct Oral Anticoagulants (DOACs) are generally preferred and recommended over warfarin due to their favorable pharmacokinetic properties, including predictable dosing, fewer dietary restrictions, and the reduced need for frequent monitoring, warfarin remains widely used, particularly in low- and middle-income countries [13,14,15]. Moreover, the optimal starting dose of warfarin remains unclear [16]. Non-pharmacological treatment options for patients who are unable to take anticoagulant therapy include surgical and endovascular occlusion of the left atrial appendage [17].

This study aimed to determine outcome measures in patients with first-ever ischemic stroke and AF regarding prior antithrombotic therapy.

## 2. Materials and Methods

This study was a retrospective cross-sectional study. Data were collected by analyzing the medical records of patients admitted to the Department of Neurology, Sveti Duh University Hospital, Zagreb, Croatia, with first-ever ischemic stroke and AF in the period from 1 January 2004 to 31 December 2013. The study was conducted in accordance with the principles outlined in the Declaration of Helsinki. Criteria for excluding subjects from the study included being under 18 years of age, having a hemorrhagic stroke, a prosthetic heart valve, anticoagulant therapy other than warfarin, antiplatelet therapy other than acetylsalicylic acid, dual antithrombotic therapy, thrombolysis and/or mechanical thrombectomy, missing values, and inadequate data. Patients receiving DOACs were excluded, as the lower prevalence of DOAC use during the study period resulted in an insufficient sample size for meaningful statistical analysis. Data collected for each patient included age, gender, risk factors, cerebrovascular risk, stroke size, and localization. The international normalized ratio (INR) value was also included in subjects on anticoagulant therapy. The risk factors analyzed were arterial hypertension, hyperlipidemia, diabetes mellitus, and cardiomyopathy. Ischemic strokes were diagnosed based on clinical examination and neuroradiological processing, including brain computerized tomography (CT) or magnetic resonance imaging (MRI). The risk of stroke development was evaluated using the CHADS_2_ scoring system (congestive heart failure, hypertension, age > 75 years, diabetes mellitus, stroke/TIA/thromboembolism), with a score of 1 point assigned for each presented risk factor [5]. Stroke severity for each subject was assessed at hospital admission using the NIHSS, based on a neurological clinical examination. The Oxford Community Stroke Project (OCSP) classification was used to assess stroke localization based on the clinical presentation of neurological deficits [18]. The size of vital cerebral tissue damage was not estimated. The subject’s level of disability and functional dependency at discharge was evaluated by the modified Rankin Scale (mRS) [19].

This study obtained approval from the Ethics Committee of the Faculty of Medicine, Osijek, and the Ethics Committee of the Sveti Duh University Hospital.

### Statistical Methods

Categorical data are represented by absolute and relative frequencies. Numerical data were described by the median and the limits of the interquartile range (IQR). Differences in categorical variables were tested with the χ^2^ test. Bonferroni’s χ^2^ adjusted residual analysis, a post hoc test, was used to test for cohort significance after obtaining a statistically significant χ^2^ test. The normality of the distribution of numerical variables was tested with the Shapiro–Wilk test. Differences in numerical variables among the three groups were analyzed using the Kruskal–Wallis test, followed by post hoc pairwise comparisons, and among two groups with Mann-Whitney U test Correlation was tested using Spearman’s rank correlation coefficient. All *p* values are two-sided. The significance level was set at Alpha = 0.05. The statistical analysis programs used were IBM SPSS Statistics for Windows (version 21.0, IBM Corp., Armonk, NY, USA).

## 3. Results

The study included 754 subjects with first-ever stroke and AF. Diagnosis of AF was previously known in 520 (69%) subjects, and 332 (44%) subjects were on prior antithrombotic therapy, of which 122 (36.7%) were taking anticoagulants, while 210 (63.3%) were taking antiplatelet therapy. Overall, the median of the NIHSS scale was 12 (IQR: 7–17), the median of the mRS scale was 5 (2–6), and the median of the CHADS_2_ grading system was 3 (IQR: 2–3).

Differences among risk factors of subjects regarding prior antithrombotic therapy are shown in Table 1. Kruskal–Wallis test and post hoc pairwise comparison of CHADS_2_ showed significant differences between the anticoagulant group and the antiplatelet group (*p* < 0.001, mean 3, IQR: 2–3, minimum 0–maximum 4 vs. mean 3, IQR: 2–3, minimum 1–maximum 4, respectively) and between the antiplatelet group and the group without antithrombotic therapy (*p* < 0.001, mean 3, IQR: 2–3, minimum 1–maximum 4 vs. mean 3, IQR: 2–3, minimum 0–maximum 4, respectively).

Among subjects with previously undiagnosed AF and without antithrombotic therapy, more than two-thirds had CHADS_2_ ≥ 2. (Table 2).

**Figure 1:**
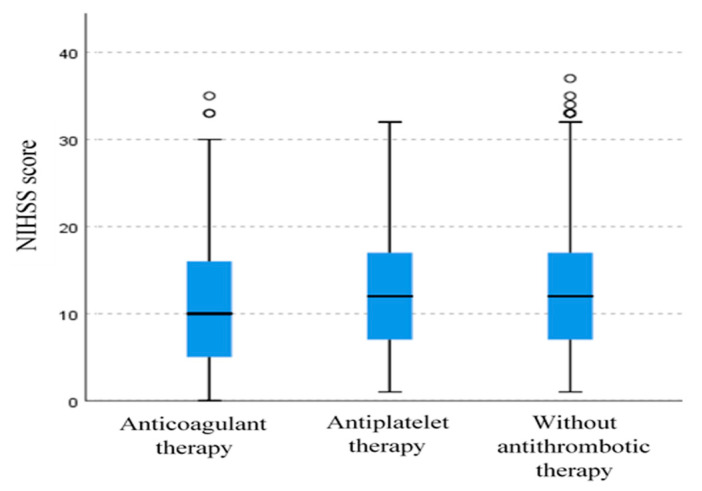
Box plot diagram (median, IQR) representing the distribution of NIHSS scores across groups of subjects with different types of prior antithrombotic therapy.

Although there was no significant difference in median NIHSS scores among the groups of subjects (the Kruskal–Wallis test, anticoagulant group vs. antiplatelet group vs. group without prior antithrombotic therapy; median 10 [IQR: 5–16] vs. 12 [IQR: 7–17] vs. 12 [IQR: 7–17]; *p* = 0.09), there is a trend toward lower NIHSS scores in the group of subjects on anticoagulant therapy (Figure 1). When compared to the anticoagulant group, there was a significant difference in the distribution of NIHSS scores among non-warfarin patients (antiplatelet group and group without antithrombotic therapy), with there being lower NIHSS scores in the anticoagulant group (Mann–Whitney U test; *p* = 0.03).

**Figure 2:**
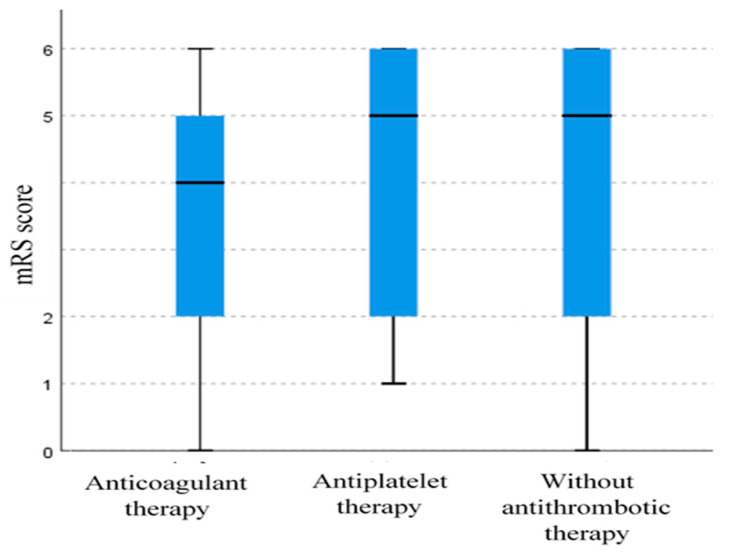
Box plot diagram (median, IQR) representing distribution of mRS scores across groups of subjects with different types of prior antithrombotic therapy.

The Kruskal–Wallis test revealed a statistically significant difference in mRS scores among the groups (*p* = 0.005). The group of subjects on anticoagulant therapy had a median mRS score of 4 (IQR: 2–5), indicating a slightly lower disability level on average compared to the other groups. A pairwise comparison showed significant differences between the group on anticoagulant therapy and the group on antiplatelet therapy. The groups of subjects on antiplatelet therapy and those without antithrombotic therapy both had a median score of 5 and an IQR of 2–6, suggesting broader variability (Figure 2). Overlapping IQRs imply variability in mRS scores across all groups, suggesting the potential influence of additional factors on subject outcomes. There is a statistically significant but weak positive correlation between the CHADS_2_ score and the mRS score (r = 0.169, *p* < 0.001).

Although the median mRS in the anticoagulant group is 4 (IQR: 2–5), a chi-square test for independence indicated that the observed count of subjects on anticoagulant therapy with mRS scores between 2 and 3 is significantly higher than in other groups. (*p* = 0.025). This suggests that subjects receiving anticoagulant therapy more often have better outcomes than groups on antiplatelet therapy and without therapy (Table 3).

## 4. Discussion

This study analyzed 754 subjects with AF and first-ever stroke, of whom more than 60% were women. The findings indicate that women with AF have a 1.75-fold higher stroke incidence than men. While previous studies have reported up to a 4.6-fold increased risk of cardioembolic stroke in women [20,21,22], the underlying cause remains unclear. These results suggest that the elevated risk is likely driven by a higher prevalence of atrial fibrosis and the prothrombotic effects of female sex hormones rather than clot formation [23]. The results of this study are consistent with a meta-analysis in which it was shown that, in about 30% of cases, the diagnosis of AF was established only during the treatment of stroke [24]. Some of these strokes could have been prevented if the diagnosis had been known, which could have been achieved by more frequent heart rhythm monitoring in at-risk subjects [25]. Almost half of the subjects with AF and CHADS_2_ score of 2 or above were without anticoagulant therapy. A possible reason for the too infrequent use of anticoagulant therapy is the existence of contraindications that were not included in this study. Another possible reason is the fear of complications, mainly bleeding, in the elderly. Previous studies show that warfarin, compared to acetylsalicylic acid, significantly reduces the risk of developing a first-ever stroke in the elderly (over 75 years of age). Elderly people also have a higher risk of developing a stroke than the risk of bleeding. That is why the benefit of anticoagulant therapy is most significant in the elderly [3,26]. Despite this, in this study, as well as in earlier studies [27], subjects on warfarin were significantly younger than the subjects on antiplatelet therapy. However, most subjects in both groups were over 70 years old. Ischemic strokes associated with AF are more severe, have a worse outcome, and have a higher mortality rate than other stroke subtypes [28,29]. In this study, the median NIHSS score was 12 (IQR: 7–17), indicating that strokes in patients with AF are often severe. This may be explained by the predominantly cardioembolic origin of strokes in AF, where the sudden occlusion of large cerebral arteries frequently leads to extensive cortical infarcts, while lacunar infarcts are much rarer. Additionally, studies have demonstrated a significant reduction in cerebral blood flow in AF patients compared to those in sinus rhythm. This diminished perfusion contributes to larger infarct areas and more pronounced neurological deficits [29,30]. Consistent with this, previous studies have reported that ischemic strokes in AF patients are frequently classified as TACI or PACI according to the OCSP classification [30,31,32]. However, in this study, no significant difference was found in OCSP classification subcategories regarding prior therapy. The observed effect of acetylsalicylic acid in reducing the incidence of LACI and POCI is likely mediated by its role in modifying lipohyalinosis of cerebral blood vessels [33].

Previous studies show significantly more severe strokes in the antiplatelet group and those without prior antithrombotic therapy, compared to subjects on prior anticoagulant therapy [34,35]. Contrary to expectations, this study reveals that the difference in severity between subjects who received prior antithrombotic therapy and those without prior antithrombotic therapy is not statistically significant; however, it does show a tendency toward lower NIHSS scores. Moreover, patients in the anticoagulant group showed a lower distribution of NIHSS scores compared to those in the other groups. The difference from the current study may be due to inadequate warfarin titration with INR outside the target values. Also, a group without prior antithrombotic therapy had significantly fewer additional comorbidities (arterial hypertension, hyperlipidemia, diabetes mellitus, and cardiomyopathy). These factors might be expected to contribute to less severe strokes. However, despite the differences in comorbidity burden, the CHADS_2_ scores between the anticoagulant group and the group without prior antithrombotic therapy did not differ significantly. Additionally, data on therapy compliance was not observed in this study. Contrary to the study conducted by Jung et al. [27], where the difference in after-stroke disability between the anticoagulant group and the antiplatelet group was not significant, in this study, the anticoagulant group shows more favorable stroke outcomes (median mRS score: 4, IQR: 2–5 vs. 5, IQR: 2–6, respectively). A potential reason for this might be that, in the study by Jung et al., patients were not categorized based on whether they had a previous stroke [27]. It is shown that when recurrent stroke adds to the existing neurological deficit or hemorrhagic transformation, patients may be more prone to complications and a worse outcome [36,37].

According to data from previous studies, anticoagulant therapy significantly reduces functional dependence after a stroke [11,38]. Significantly lower median mRS scores were observed in the anticoagulant group compared to the antiplatelet group. Significantly higher observed frequencies of mRS scores 2 and 3 were noted in the anticoagulant group compared to both the antiplatelet group and the group without antithrombotic therapy, suggesting a trend toward better outcomes in the anticoagulant group and its superiority over antiplatelet therapy. The results of some studies show that antiplatelet therapy does not lead to a better outcome nor reduce the severity of stroke. Antiplatelet therapy significantly reduces the severity and outcome of strokes, but only in those caused by atherosclerosis of blood vessels [38,39,40]. Other studies show that prior use of antiplatelet therapy may still improve functional stroke outcome, but it is not better than prior anticoagulant therapy [12]. Since people with AF usually also have vascular diseases, the effect of antiplatelet therapy in improving the functional outcome is probably indirect and mediated by the impact on atherosclerotic vascular disease [41].

One limitation of this study is its cross-sectional design, which restricts the ability to infer causality. In this study, we also did not assess the severity of individual comorbidities, which could have varying effects on stroke severity. A comparison with neurological findings, which include the size of the stroke, was not conducted either. Due to the retrospective cross-sectional study design, we were unable to include existing contraindications for antithrombotic therapy or indications for initiating therapy.

## 5. Future Directions

Further research with a larger sample size and a longitudinal design is needed to better understand how comorbidity burden and prior antithrombotic therapy influence stroke severity. Future studies could also investigate how the duration of antithrombotic therapy affects stroke outcomes, or other treatment options, such as early rhythm control, may influence the outcome of these patients [42]. The role of screening for atrial fibrillation can be improved by utilizing modern technology, which may aid in developing more accurate stroke prediction models [43]. Research and clinical trials have shown that DOACs are not efficacious in some clinical scenarios where warfarin is still useful in stroke prevention (e.g., valvular atrial fibrillation, mechanical heart valves, embolic strokes of undetermined source, or antiphospholipid syndrome) [44].

## 6. Conclusions

Our study showed that first-ever ischemic stroke due to atrial fibrillation was more common in women in our cohort. Subjects on prior anticoagulant therapy (warfarin) had more favorable outcomes at discharge.

## Figures and Tables

**Table 1:** Differences in characteristics of subjects according to risk factors and stroke localization to type of prior antithrombotic therapy.

Characteristics		Anticoagulant Therapy (a)	Antiplatelet Therapy (b)	Without Antithrombotic Therapy (c)	*p* *
Gender [n (%)]					
female		76 (62.3)	128 (61)	276 (65.4)	0.52
male		46 (37.7)	82 (39)	146 (34.6)	
Age [median, (IQR)]		76 (71–81)	80 (75–84)	80 (74–84)	0.001 ^†^
Arterial hypertension [n (%)]		112 (91.8)	196 (93.3)	359 (85.1)	0.004 ^‡^
Hyperlipidemia [n (%)]		67 (54.9)	97 (46.2)	167 (39.6)	0.008 ^§^
Diabetes mellitus [n (%)]		42 (34.4)	83 (39.5)	121 (28.7)	0.02 ^||^
Cardiomyopathy [n (%)]		89 (73)	159 (75.7)	265 (62.8)	0.002 ^¶^
CHADS_2_ score [median (IQR)]		3 (2–3)	3 (2–3)	3 (2–3)	<0.001 **
OCSP	TACI	24 (19.7)	36 (17.1)	48 (11.4)	0.116
	PACI	51 (41.8)	105 (50)	225 (53.3)	
	LACI	24 (19.7)	38 (18.1)	72 (17.1)	
	POCI	23 (18.9)	31 (14.8)	77 (18.2)	

IQR, interquartile range; OCSP, Oxfordshire Community Stroke Project; TACI, total anterior circulation infarct; PACI, partial anterior circulation infarct; LACI, lacunar circulation infarct; POCI, posterior circulation infarct; * χ^2^ test; ^†^ Kruskal–Wallis test (pairwise comparison) of significant differences ((a) vs. (b) and (a) vs. (c)); ^‡^ adjusted residuals 1.3 (a), 2.6 (b), −3.3 (c); the significant adjusted *p*-value *p* = 0.009 (b), *p* = 0.001 (c); ^§^ adjusted residuals 2.7 (a), 0.8 (b), −2.7 (c); the significant adjusted *p*-value *p* = 0.007 (a), *p* = 0.007 (c); ^||^ adjusted residuals 0.5 (a), 2.5 (b), −2.6 (c); the significant adjusted *p*-value *p* = 0.012 (b), *p* = 0.009 (c); ^¶^ adjusted residuals 1.3 (a), 2.8 (b), −3.5 (c); the significant adjusted *p*-value *p* = 0.005 (b), *p* < 0.001 (c); ** Kruskal–Wallis test (pairwise comparison); the significant difference (a) vs. (b) and (b) vs. (c).

**Table 2:** Distribution of subjects regarding CHADS_2_ rating scale in relation to type of prior antithrombotic therapy and previously known atrial fibrillation.

Previous AF	[n (%)]	CHADS_2_ ≤ 1	CHADS_2_ ≥ 2
Unknown	Anticoagulant therapy	1 (2.6)	1 (0.5)
	Antiplatelet therapy	1 (2.6)	48 (24.5)
	Without antithrombotic therapy	36 (94.7)	147 (75)
Known	Anticoagulant therapy	13 (25.5)	213 (45.4)
	Antiplatelet therapy	12 (23.5)	149 (31.8)
	Without antithrombotic therapy	26 (51)	107 (22.8)

AF—atrial fibrillation.

**Table 3:** Differences in stroke outcome based on mRS scale across groups of subjects with different types of prior antithrombotic therapy (mRS, modified Rankin scale).

mRS [n (%)]	Anticoagulant Therapy (a)	Antiplatelet Therapy (b)	Without Antithrombotic Therapy (c)	*p* *
mRS ≤ 1	17 (13.9)	18 (8.6)	50 (11.8)	
Adjusted residual	1.0	−1.5	0.6	
2 ≤ mRS ≤ 3	43 (35.2)	43 (20.5)	104 (24.6)	
Adjusted residual	2.8	−1.9	−0.4	0.025
4 ≤ mRS ≤ 5	34 (27.9)	80 (38.1)	153 (36.3)	
Adjusted residual	−1.9	1.0	0.5	
mRS = 6	28 (23)	69 (32.9)	115 (27.3)	
Adjusted residual	−1.4	1.8	−0.6	

* χ^2^ test.

## Data Availability

The data presented in this study are only available upon request to the corresponding author for ethical reasons.
